# Cryo-electron Microscopy Structure of the Native Prototype Foamy Virus Glycoprotein and Virus Architecture

**DOI:** 10.1371/journal.ppat.1005721

**Published:** 2016-07-11

**Authors:** Grégory Effantin, Leandro F. Estrozi, Nick Aschman, Patricia Renesto, Nicole Stanke, Dirk Lindemann, Guy Schoehn, Winfried Weissenhorn

**Affiliations:** 1 Institut de Biologie Structurale (IBS), Univ. Grenoble Alpes, CEA, CNRS, Grenoble, France; 2 UVHCI, Univ. Grenoble Alpes, CNRS, EMBL, Grenoble, France; 3 Univ. Grenoble Alpes, TIMC-IMAG, Grenoble, France; 4 CNRS, TIMC-IMAG, Grenoble, France; 5 Institute of Virology, Medical Faculty “Carl Gustav Carus”, Technische Universität Dresden, Dresden, Germany; Universitätklinikum Heidelberg, GERMANY

## Abstract

Foamy viruses (FV) belong to the genus Spumavirus, which forms a distinct lineage in the *Retroviridae* family. Although the infection in natural hosts and zoonotic transmission to humans is asymptomatic, FVs can replicate well in human cells making it an attractive gene therapy vector candidate. Here we present cryo-electron microscopy and (cryo-)electron tomography ultrastructural data on purified prototype FV (PFV) and PFV infected cells. Mature PFV particles have a distinct morphology with a capsid of constant dimension as well as a less ordered shell of density between the capsid and the membrane likely formed by the Gag N-terminal domain and the cytoplasmic part of the Env leader peptide gp18^LP^. The viral membrane contains trimeric Env glycoproteins partly arranged in interlocked hexagonal assemblies. *In situ* 3D reconstruction by subtomogram averaging of wild type Env and of a Env gp48^TM^- gp80^SU^ cleavage site mutant showed a similar spike architecture as well as stabilization of the hexagonal lattice by clear connections between lower densities of neighboring trimers. Cryo-EM was employed to obtain a 9 Å resolution map of the glycoprotein in its pre-fusion state, which revealed extensive trimer interactions by the receptor binding subunit gp80^SU^ at the top of the spike and three central helices derived from the fusion protein subunit gp48^TM^. The lower part of Env, presumably composed of interlaced parts of gp48^TM^, gp80^SU^ and gp18^LP^ anchors the spike at the membrane. We propose that the gp48^TM^ density continues into three central transmembrane helices, which interact with three outer transmembrane helices derived from gp18^LP^. Our ultrastructural data and 9 Å resolution glycoprotein structure provide important new insights into the molecular architecture of PFV and its distinct evolutionary relationship with other members of the *Retroviridae*.

## Author Summary

Foamy viruses (FVs), which belong to the retroviral genus Spumavirus, are endemic to non-human primates and can be transmitted to humans. They are considered as potential vectors for gene therapy due to their broad cell tropism and their apparent apathogenicity in natural hosts and humans. In order to gain more insight into the ultrastructure of the prototype FV (PFV) we performed (cryo-)electron tomography and microscopy of infected cells and of isolated virions. We find that PFV contains a nucleocapsid of constant dimensions at its center, an intermediate shell of protein positioned between the core capsid and the viral membrane and glycoprotein that arranges into regular hexagonal lattices on the virus membrane. Structural analysis of the glycoprotein was performed *in situ* to a resolution of 9Å, which shows regular helical features such as a trimeric coiled coil of the fusion protein subunit, a hallmark of class I fusion proteins, spacer arms between the glycoprotein trimers and the arrangement of six transmembrane helices, a characteristic feature of the PFV Env glycoprotein. We discuss our results in light of the evolutionary relationship of PFV with other retroviruses as well as the role of the unique glycoprotein architecture on the virus life cycle.

## Introduction

Spuma or foamy viruses (FV) are the only members of the *Spumaretrovirinae* subfamily of the *Retroviridae*. As such they share many similarities in their life cycle with the *Orthoretrovirinae* as well as some features with the more distant *Hepadnaviridae *[1]. FVs infect nearly all mammals and the best-studied member is the Prototype FV (PFV) previously called Human FV (HFV), which has been isolated from infected human cells [2]. FV infection in humans is asymptomatic [3] but the virus can replicate very efficiently in human cell lines and is therefore an elegant model system for the study of more hazardous orthoretroviruses and a promising candidate for gene transfer therapy [4].

The main PFV structural protein is Gag (648 amino acids (aa)), which is encoded as a single protein and unlike orthoretroviruses does not exist as a Gag/Pol fusion variant. PFV Gag is also unusual in that it is not processed by the viral protease into canonical Matrix (MA), Capsid (CA) and Nucleocapsid (NC) domains, like other retroviral Gag polyproteins. The 71 kDa PFV Gag (pr71^Gag^) precursor is only partially proteolysed at its C-terminus to yield a 68 kDa (p68^Gag^) and a 3 kDa (p3^Gag^) peptide [5,6]. In addition, three less systematic secondary cleavage sites are located in the middle of the Gag sequence (residues 311, 339 and 352) and have been proposed to serve capsid disassembly during virus entry [5,7]. The C-terminus of PFV Gag contains a Glycine–Arginine Rich (GR) region, which is important for interaction with the viral RNA genome and is the functional equivalent to the Cys-His motif found in orthoretroviruses [8]. PFV Gag lacks a membrane-binding domain and instead virus egress relies on a physical interaction between Gag and the Leader Peptide (LP) domain of the Envelope (Env) protein [9,10]. The PFV Env precursor comprises 988 residues and undergoes post-translational processing by cellular proteases resulting in three distinct domains: Surface (SU) gp80^SU^, TransMembrane (TM) gp48^TM^ and LP gp18^LP^ [11]. Mutants defective in the SU/TM cleavage produce non-infectious virions [11,12] while an inactive SU/LP processing inhibits budding [13]. Env forms trimers of heterotrimers (gp80-gp48-gp18) anchored by two transmembrane regions (per monomer) in the virus membrane, where it can further assemble into hexameric lattices [14]. PFV capsid assembly commences at the centrosome or microtubule organizing center similar to type B/D orthoretrovirus assembly [15]. Capsids are then transported to the secretory pathway either the ER or the Golgi or directly to the plasma membrane where Env LP interacts with the N-terminal Gag region [9,10,16]. Budding into intracellular compartments or at the plasma membrane depends on the recruitment of the ESCRT machinery [17,18], which completes budding by membrane scission [19]. Intracellular virions are most likely transported in vesicles for release at the plasma membrane (reviewed in [1]).

In order to obtain structural insight into PFV organization at medium resolution we used cryo-electron tomography (cryo-ET) and microscopy (cryo-EM) to analyze isolated wild-type particles and variants with mutations in PFV Gag and Env proteins. These data are correlated to Gag assemblies analyzed in infected cells by employing high pressure freezing, cryo-substitution and electron tomography. We further present a 9 Å resolution structure of the glycoprotein *in situ*, which shows unprecedented molecular details of its membrane-anchored organization and higher order assemblies stabilized most likely by the leader peptide gp18^LP^.

## Results

### *In vivo* study of PFV infected cells

**Fig 1:**
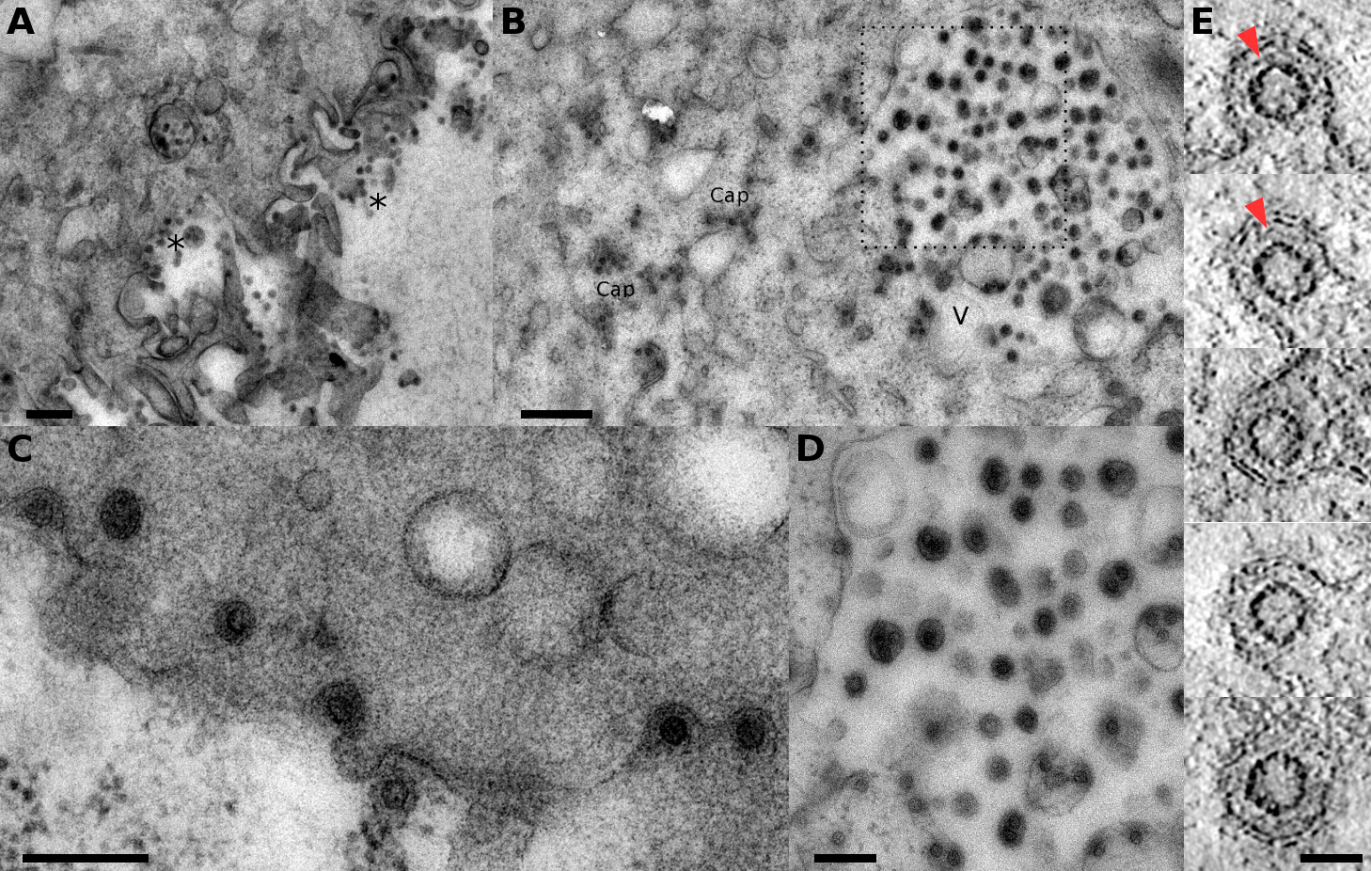
Analysis of PFV infecting HT1080 cells. A, B- Low magnification view of PFV infected cells showing budding at the plasma membrane (indicated by * symbol) (A) and viruses in intracellular vacuoles (symbol V) (B). Free (non-enveloped) cytoplasmic capsids can be seen as well (labeled Cap in (B)). C, D- Higher magnification view of viruses budding at the plasma membrane (C) and viruses possibly budding in vacuole (D–zoom in the rectangular region of (B)). E- 0.8 nm thick tomogram slice through five different viruses budding at the plasma membrane. The red arrowheads point to the intermediate shell between the capsid and the plasma membrane. Scale bars in A—B, C—D and E are 500, 200 and 60 nm respectively.

In order to probe the various structures of PFV found *in vivo* and correlate the results with the studies on purified particles analyzed by cryo-ET (see below), we prepared samples of HT1080 cells infected with replication competent wt PFV by high pressure freezing and cryo-substitution 24 to 48h post infection (see Material and Methods). Viruses with clear capsid at their center were observed budding from the plasma membrane (Fig 1A and 1C). Viruses were often found as well into large vacuolar compartments which can either be related to plasma membrane budding or constitute an alternative budding site (Fig 1B and 1D). Naked capsids were also observed in the cytoplasm (Fig 1B). All these observations agree well with previous results obtained in [15]. Electron tomography of 100 to 300 nm thick sections of viruses budding from the plasma membrane (Fig 1E) show that the virions are composed of the capsid spaced from the viral membrane by an additional fainter intermediate shell of density (Fig 1E, arrowheads). In addition to the previous observations, we found on several occasions cells with an unusually high concentration of round objects in the cytoplasm (S1 Fig), which are rather regular in size (r = 28 nm) similar to the capsid size of released virions and the one observed at the plasma membrane (S1 Fig). They are also often aligned in membrane delimited tubes (S1 Fig and S1 Movie). Because, we did never observe such assemblies in non-infected HT1080 cells, we speculate that they constitute assembled capsids, which accumulate in tubular membrane compartments.

### Preparation of wt and mutant PFVs for cryo-ET and cryo-EM

Three different PFV viruses, wild type virus (wt), a Gag mutant impaired in RNA binding (iNAB) and an Env mutant (iFuse) were purified from the supernatant of cells expressing different combinations of PFV proteins from a replication-deficient PFV vector system. In the iNAB mutant, 23 arginines in the glycine/arginine rich (GR) region in the C-terminus of Gag have been replaced by alanine, which results in a Gag protein unable to bind nucleic acid [20] (S2 Fig). Virus particles are still released from cells, although less efficiently, but are non-infectious and display capsid assembly defects. The iFuse mutant is a variant of Env where the furine cleavage site between the SU (gp80^SU^) and TM (gp48^TM^) domains of Env has been mutated [11,12] (S2 Fig). This results in a partially processed glycoprotein as the cleavage between the LP and SU domains is preserved. Particles are released at nearly wild type level from cells but are non-infectious. The presence of viral proteins of wild type and mutant viruses (pr71^Gag^, p68^Gag^, gp130^Env^ for the iFuse mutant, gp80^Env^ and gp18^LP^ for wt and iNAB mutant) were confirmed by Western blot analysis (S2 Fig).

### Cryo-electron tomography of purified PFV virus

**Fig 2:**
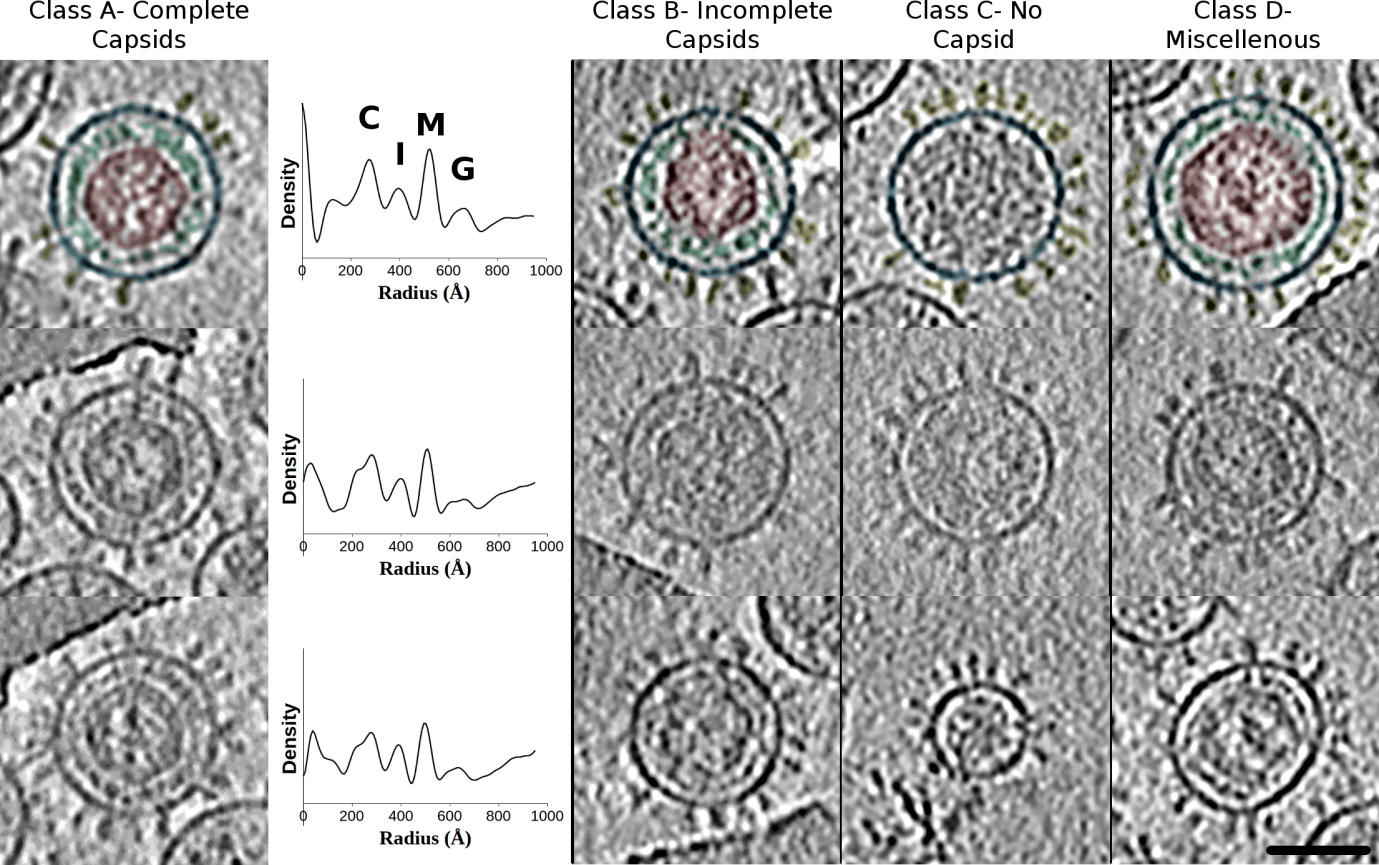
Cryo-electron tomography of wt PFV. Central slices through wt PFV virions having a complete capsid (Class A), an incomplete capsid (Class B), no capsid (Class C) or miscellaneous particles (Class D). For each class, one virus has its capsid, intermediate shell, viral membrane and glycoproteins colored in red, green, blue and yellow respectively. The interior of Class C virus having no characteristic features is left uncolored. For each class A virus, the corresponding radial density profile is shown and the various peaks corresponding to the glycoprotein (G), viral membrane (M), intermediate shell (I) and capsid (C) are labeled on one plot. Scale bar is 60 nm.

When observed by cryo-ET, PFV wt forms mainly near spherical particles (Fig 2) as previously reported in [16]. The Env glycoprotein projections on the virus surface are ~14 nm in length. Underneath the membrane a fuzzy density not directly attached to the membrane connects to the capsid. Rare oval shaped viruses also exist but no tubular, elongated or more irregular structures were observed (Fig 2). Although the majority of viruses are spherical, their overall sizes are variable, ranging from 28 to 63 nm in radius measured from the center of the particle to the outer margin of the viral membrane (r_mean_ = 45.6 ± 8.2 nm (n = 141)) confirming previous observations [16]. Apart from their dimensions, PFV particles differ with respect to their internal structures. We classified PFV wt particles into four main classes (A to D) according to the variation in shape and morphology of their interior (Fig 2). Class A virus (15% of total particles) contains an intact core at the center of the particle (r_mean_ = 30.0 ± 0.6 nm (n = 25)) consistent with previous measurement [16]. The capsid is surrounded by a second shell of density (width~ 5–6 nm), which most likely corresponds to the Gag N-terminal domain that interacts with Env LP [10,16]. Class B (20% of total particles) is similar to A except that the central Gag core is either incomplete or disrupted resulting in an open Gag structure, which generally merges with the intermediate shell. Class C (45% of total particles) contains spherical particles with no regular, ordered internal structures and class D (20% of total particles) miscellaneous particles having non spherical shape or an interior morphology significantly different from class A and B. We interpret classes A, B and (most of) D as particles containing capsids. Such viruses have been observed by electron microscopy of infected cells (Fig 1) [15,21] and by cryo-EM [16,22]. They likely represent the released, mature and infectious form of PFV. Whether any of the virions of class C are infectious or not remains to be determined [23,24].

**Fig 3:**
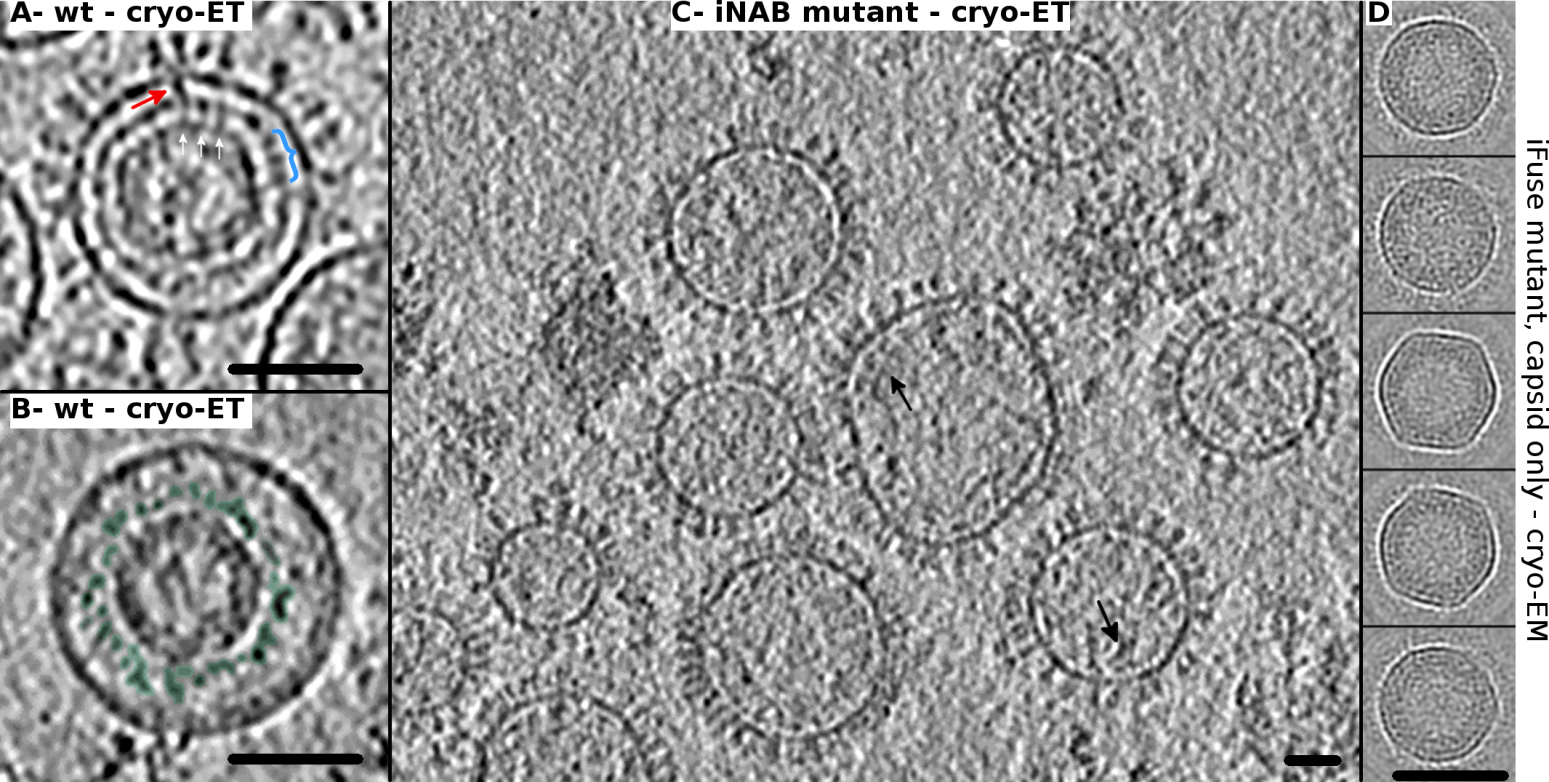
Ultrastructure of PFV virus interior. A- Central slice through a wt PFV virus imaged by cryo-ET. The white and red arrows point to some of the interactions/connections between capsid / intermediate shell and intermediate shell / viral membrane respectively. The blue parenthesis highlights local organization in the intermediate shell. B- Same as (A) except that this virus has no glycoprotein sticking out of the membrane but it still has a capsid with an intermediate shell (colored in green). C- Tomogram section of PFV iNAB mutant showing viral particles without any internal ordered structure. Black arrows point internal densities located close to the viral membrane but distinct from the intermediate shell of wt and iFuse PFV. D- 2D classification on the capsid alone of iFuse mutant viruses imaged by cryo-EM. Five 2D class averages are shown which could be interpreted as 2D projections of a pseudo-icosahedral object viewed along the 5, 5, 3, 2 and 5-fold symmetry axes respectively. Scale bars are 60 nm for each panel.

For near spherical viruses (classes A to C), the distribution of particle dimensions within each group is different. Class A virions are less variable in dimensions (r_mean_ = 54.1 ± 2.9 nm, n = 25) than the other classes (r_mean_ = 50.3 ± 5.2 nm, n = 35 and 40.7 ± 6.7 nm, n = 81 for class B and C respectively) (S3 Fig). This implies that a complete capsid with the intermediate shell leads to particles with more homogeneous dimensions. Although the viral membrane, capsid and intermediate shell appear as distinct (separated) substructures in tomograms, there are nevertheless clear interactions between them (capsid/intermediate shell and intermediate shell/membrane bilayer) (Fig 3A) as indicated previously [16], which probably contribute to the stability of the virus and the narrower size distribution of Class A viruses. When complete, the capsid at the center of particles has a relatively uniform mean radius (r ~ 30 nm) (Fig 2, class A–radial plots) but few exceptions with larger and less uniform shapes exist as well (Fig 2, class D). The population with a constant radius often has a hexagonal outline with more or less sharp vertices by cryo-ET. *Ab initio* 2D class averages of only the capsid, calculated from images of iFuse mutant (identical to wt in term of Gag sequence) acquired by cryo-EM, reveal a variety of capsid shapes ranging from hexagonal to near round (Fig 3D). This could be an indication of a regular, (pseudo-)symmetrical arrangement of the capsid. The capsid shell is relatively thick (~120 Å) as there are strong densities associated with the capsid outer wall while its center appears comparatively less dense (Fig 2, plots and Fig 3D). The intermediate shell, which strictly follows the contour of the capsid, precludes a direct interaction of the capsid with the viral membrane. This 5–6 nm thick shell is less polyhedral (hexagonal) and appears discontinuous and structurally variable. Despite that, local regular organization (spacing ~ 8–9 nm) are visible in some tomographic slices (Fig 3A) [22].

The iNAB mutant can't bind the viral RNA or any nucleic acid. We previously showed by cryo-EM that this mutant displays severe assembly defects [20]. In the present study, we extended the analysis of iNAB mutant to cryo-ET to unambiguously visualize the interior of the viral particles (Fig 3C). As wt PFV, the iNAB mutant forms a majority of near spherical particles on which the glycoproteins are clearly visible. Their dimensions (r_mean_ = 53.1 ± 10.3 nm, n = 42) are larger than wt PFV (r_mean_ = 45.6 ± 8.2 nm). On the average, more deformed and irregular particles are observed compared to wt PFV. We also confirm our previous result that without the RNA binding motif in Gag, and hence no RNA interaction, no regular capsid are assembled inside the near spherical particles (Fig 3C). However, these particles are not empty and some diffuse densities are detected often in the vicinity of the viral membrane (Fig 3C–arrows) but they are distinct from the intermediate shell visualized in wt PFV. We conclude that the capsid and the intermediate shell observed for wt and iFuse PFV (S3 Fig) are mainly resulting from a co-assembly of Gag with viral RNA to form the nucleocapsid. The identification in some wt PFV tomogram of sections that reveal a capsid plus the intermediate shell and no glycoprotein on the surface strengthens this conclusion (Fig 3B) as it rules out the possibility that the intermediate shell is made only of the Env gp18^LP^ cytoplasmic domain. Nevertheless, the Env gp18^LP^ cytoplasmic domain (67 residues long) has been shown to interact with the N-terminal domain of Gag [10] and therefore it most likely contributes as well to the overall intermediate shell density of mature PFV particles (Class A, B and D of Fig 2).

### 3D structure of PFV glycoprotein by cryo-ET

**Fig 4:**
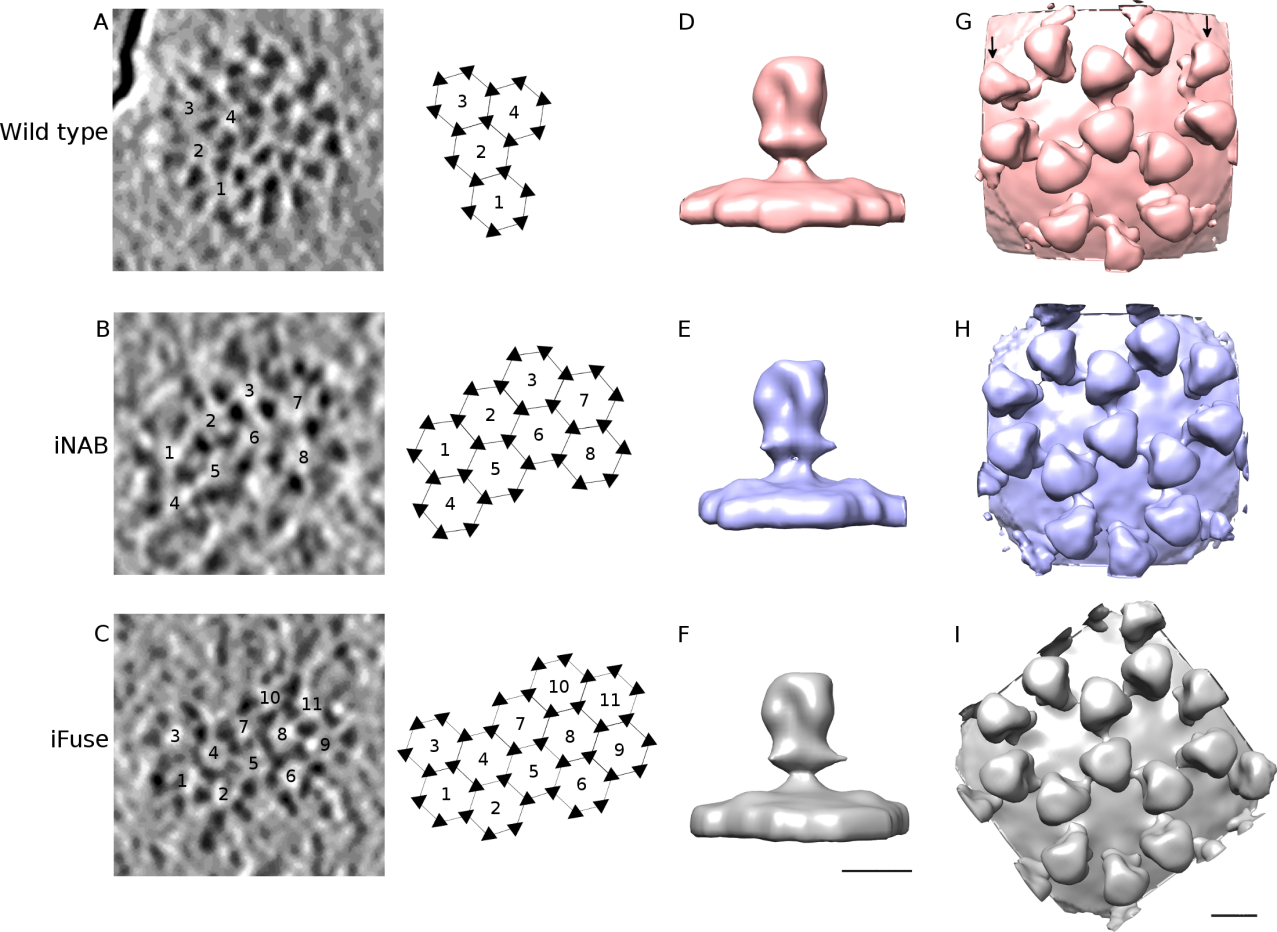
Subtomogram averaging of PFV glycoprotein. A–C- 0.8 nm thick tomographic slice perpendicular to the glycoprotein long axis from the wt, iNAB and iFuse mutants and its corresponding schematic of interlocked hexagonal assemblies of trimers. Numbers are indicated at the center of each hexagon and triangles represent the position of each trimer of Env in the hexagonal network. The wt virus shows ordered region of glycoprotein (represented on the corresponding schematic on the right) next to less ordered one while both mutants have more ordered hexagonal networks. D–I- Subtomogram averaging on glycoproteins of wt (top), iNAB (middle) and iFuse (bottom) mutants. D–F and G–I show a side view of a single trimer and a top view of intertwined hexagonal assemblies respectively. The two arrows in (G) point to less well defined peripheral trimers on wt particles. Scale bars are 100 Å.

Wt PFV and the iNAB mutant particles should have an identical Env structure (same amino acid sequence, same processing by proteases), at least for the extracellular domain. The iFuse mutant lacks the gp80^SU^-gp48^TM^ cleavage, which could influence the position of the fusion peptide region, but should have otherwise a similar structure than wt. Central slices through tomograms of the samples show that the glycoprotein extends around 14 nm away from the membrane while sections perpendicular to the glycoprotein's long axis clearly confirm that they are trimeric (S3 Fig) [14]. The 3D reconstruction of PFV wt, iNAB and iFuse glycoproteins by subtomogram averaging at ~3 nm resolution (S3 Fig) demonstrates that all three structures have a similar knob-like shape at this resolution (Fig 4D–4F). They are arranged such that each trimer can interact with up to three other trimers to form a network of interlocked hexagons (Fig 4A–4C and 4G–4I). This hexagonal network organization described previously in [14] is not obligatory as the viral membrane is not fully covered with glycoproteins. Isolated trimers were hardly found but incomplete hexagonal networks (with less than six trimers) were observed. Less ordered areas are also possible as well as pentagonal arrangements (Fig 4A and S3 Fig). From cryo-ET reconstructions of a network of three adjacent and interlocked hexagons for wt PFV (Fig 4G), it appears that the trimers at the periphery (arrows in Fig 4G) are less well defined than the central one, which indicates that the occupancy rate of the trimers within hexagons is quite low or that not all glycoproteins arrange in a hexagonal fashion or that the hexagonal packing is flexible. On the contrary, the same reconstructions of iNAB and iFuse mutants (Fig 4H and 4I) show stronger resolved features for all trimers, including the one at the periphery. This was also confirmed directly on tomograms where slices orthogonal to the mutant's glycoprotein long axis often display more regular hexagonal lattices than the wt (Fig 4B and 4C). Thus, it appears that the two mutants have a stronger propensity to form a regular hexagonal network suitable for 3D reconstruction than the wt. It is not clear if this observation results directly from the mutations of the iNAB and iFuse samples or if the observed difference simply results from variability between virus preparations.

### 3D structure of PFV glycoprotein by cryo-EM

**Fig 5:**
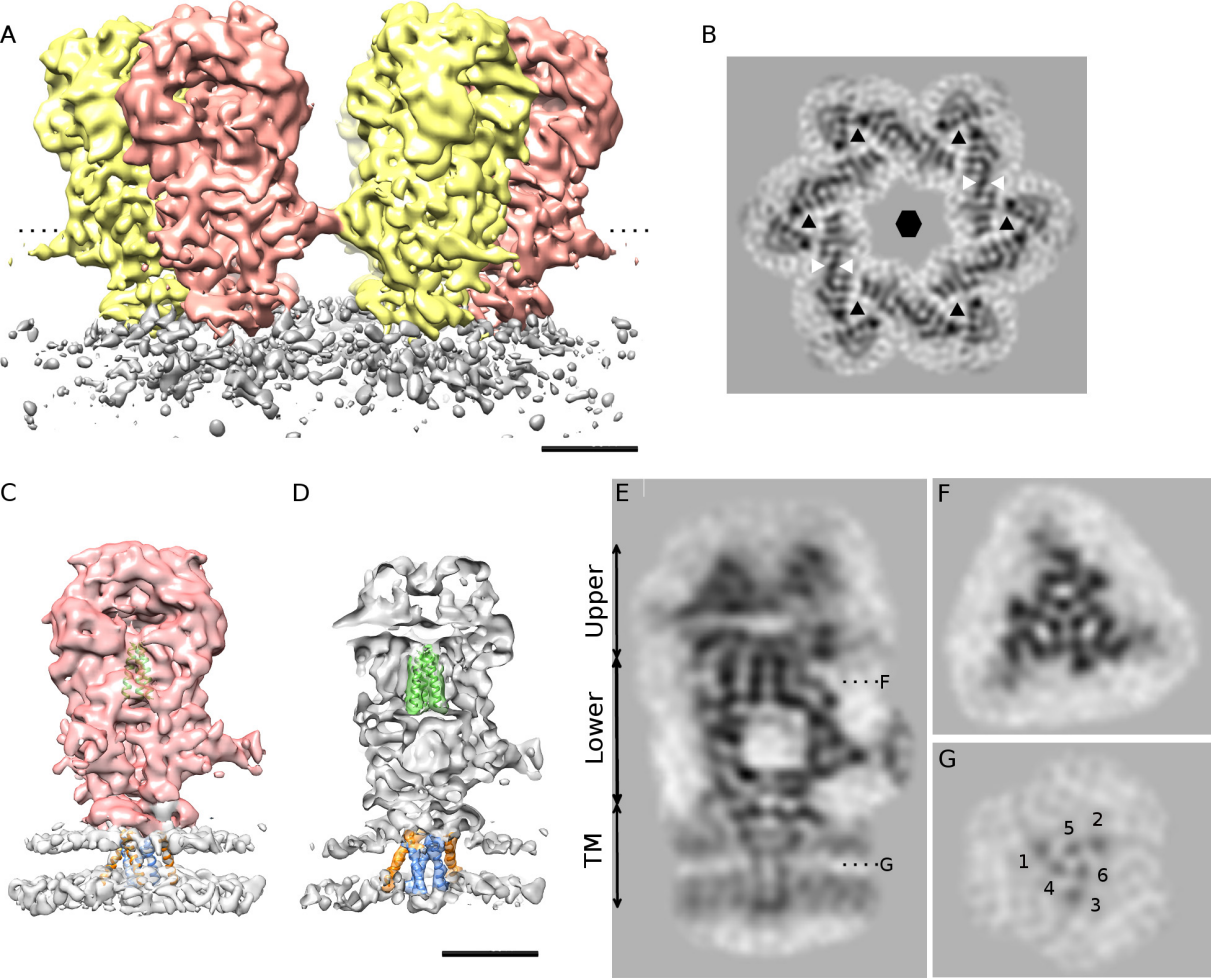
*In Situ* single particle 3D reconstruction of PFV glycoprotein by cryo-EM. A- Side view of the sharpened 3D reconstruction of an hexagonal assembly of trimeric glycoproteins from the iFuse mutant (6-fold symmetry applied, ~10 Å resolution at FSC = 0.143). Spikes are colored alternately yellow and salmon, viral membrane is gray. B- Gray scale section at the level of the dotted line in (A). The triangle and hexagon symbols indicate the position of each trimer and of the 6-fold axis orthogonal to the section plane respectively. The two white arrowheads delimit the region where the trimers are interacting with each other. C, D- Side views (full (C) and cut-away (D) views) of a single PFV Env trimer (sharpened map) after 3-fold symmetry application (~9 Å resolution at FSC = 0.143). The densities corresponding to the extracellular domains and the viral membrane are colored salmon and gray respectively in C. The three central helices attributed to gp48 fusion peptide are represented by three green α helices of 22 residues long each. The TMHs are represented by three inner (colored blue) and three outer (colored orange) α helices. In D, the densities surrounding the three central helices and the three inner and outer TMHs are colored green, blue and orange respectively while the remaining of the spike is gray colored. E, F and G- Grayscale sections through the reconstruction shown in (C): E- central side view as in (D), F and G- top views at the level of the dotted lines in (E) through the three helix bundle and the TMHs respectively (the three outer and inner TMHs are labeled 1, 2, 3 and 4, 5, 6 respectively). Scale bars are 50 Å in A, B, D, E.

To gain more insights into the structure of the PFV glycoprotein, we use 3D reconstructions of iNAB and iFuse Env hexagonal assemblies of six trimers determined by subtomogram averaging as initial references for automatic particle selection of micrographs acquired by cryo-EM (S4 Fig) (see Material and Methods). The data were refined to higher resolution imposing C6 symmetry. Once the refinement converged, the six equivalent trimers of the hexagonal assembly were 3-fold symmetrized to yield the final single spike structure. The iNAB Env (a fully processed Env as in wt virus) and the iFuse mutant (a gp80^SU^-gp48^TM^ cleavage site mutant) were computationally processed independently but yielded virtually identical structures at ~9 Å resolution at FSC = 0.143 (S4 Fig). In the density map of the hexagonal assembly (Fig 5A and 5B), the neighboring trimers are spaced by ~110 Å and are interacting with each other approximately 45 Å above the membrane level. This interaction seems to serve as a spacer for hexagonal lattice formation (Fig 5A and 5B). There is no sign of ordered densities extending from the inner leaflet of the membrane towards the virus interior, which can be attributed to the cytoplasmic domain of Env gp18^LP^ or the N-terminal region of Gag. The PFV Env trimer can be decomposed in an upper and lower region followed by the transmembrane (TM) region (Fig 5C–5E). The upper part likely composed of gp80^SU^ forms three arch-like structures, which join at the top and delineate a less dense area in its center (Fig 5E). We propose that the three central short rods of density visible at the top of the lower part and surrounded by extra density are three α-helical regions derived from the fusion protein subunit gp48^TM^ (Fig 5C–5F). Gp48^TM^ contains two potential coiled coil regions from residues 664 to 685 and 881 to 902 [25] or residues 664 to 691 and 880 to 908 [26], which can correspond to heptad repeat region 1 and 2, respectively, present in retroviral class I fusion proteins [27]. Based on the cryo-EM map, the length of the predicted helices is approximately 32 ± 5 Å (Fig 5C–5E), which is consistent with 6 to 7 helical turns as predicted by sequence analysis. However, due to the limited resolution of the reconstruction, the actual helices may be slightly longer. The lower part of the spike is likely composed of parts of gp80^SU^ and gp48^TM^, which anchor the extracellular domain in the membrane (Fig 5C and 5D). The interaction on the top via the coiled coil and the one close to the membrane generate a central open space (Fig 5C–5E and S5 Fig). Each Env monomer is predicted to have two transmembrane helices (TMH): one in the gp18^LP^ domain between aa 68–89 and one in the gp48^TM^ domain between aa 961–980. We propose that the gp48^TM^ density extends into three central TMHs, which interact with three outer TMHs derived from the gp18^LP^ (Fig 5C, 5D and 5G). Notably, the three central TMHs of gp48^TM^ are positioned such that they can interact with each other. Furthermore each TMH derived from gp18^LP^ is in close contact to one gp48^TM^ TMH (Fig 5C, 5D and 5G). However, at the current resolution of the map, we cannot exclude the possibility that the three central helices are derived from gp18^LP^ and the external ones from gp48^TM^

## Discussion

### Spumavirus and orthoretrovirus assembly

The majority (~80%) of wt PFV virions have a nearly spherical shape with an average radius of ~54 nm as indicated previously in [16] which is smaller than mature particles formed by other orthoretroviruses such as HTLV-1 (57 nm) [28], HIV-1 (73 nm) [29,30] and Rous Sarcoma Virus (63 nm) [31]. Moreover, PFV viruses containing an intact capsid (class A) have an even more uniform radius. PFV budding is strictly dependent on the physical interaction between Env and Gag [21]. As Gag with RNA forms internal structures (nucleocapsid with a surrounding shell) of homogeneous size, it is not surprising that the viral membrane that contains inserted Env trimers ends up near spherical as well and follows closely the contour of the Gag assembly. Therefore, the narrower size distribution of PFV compared to other retroviruses could be the direct consequence of the interaction of Env with a preformed nucleocapsid.

We interpret the viruses containing well-defined internal Gag structures (class A, B and D of Fig 2) as the mature PFV particle. This is supported by *in vivo* observation of the same viral layout in virus infected cells. This organization is quite different from the mature particles of other retroviruses, which consist of a polyhedral core of CA containing the NC/RNA complex [28,30,31] as well as from their immature forms [32–35]. These differences in virus assembly likely relate directly to differences in posttranslational processing. Gag from most retroviruses is cleaved after the release of immature virions from the cell at various positions, which triggers the reorganization of the MA, CA and NC domains inside the virus [36]. For PFV, there is only one primary cleavage of Gag by the viral protease occurring in the course of virus assembly in infected cells, which produces the two Gag products p68^Gag^ and p3^Gag^ [5,6].

PFV virions contain an extra layer of intermediate density between the viral membrane and the capsid which we believe is derived from the Gag N-terminal domain and the cytoplasmic domain of the gp18^LP^ as suggested previously [16]. This also indicates that in mature virions, the Gag N-terminal and gp18^LP^ domains are flexibly linked to the capsid core and the glycoprotein respectively. Secondary cleavages have been also observed at three different positions of Gag [5], however, their relevance for the virus life cycle needs to be established.

### PFV and Hepadnaviridae similarities

Foamy viruses also share similarities with Hepatitis B virus. Notably HBV contains a capsid with icosahedral symmetry twice smaller (r = ~14 nm) than PFV [37], buds intracellularly in the ER and forms large amounts of capsidless subviral particles used as a decoy for the host immune system [38]. Although PFV capsids can be variable in morphology, the majority has a uniform mean radius of 30 nm with a round to hexagonal outline. The latter are compatible with 2D projections of a symmetrical object including icosahedra. However, the analysis of intact capsids by either cryo-ET or cryo-EM did not allow defining a clear repetitive unit. The same conclusion was obtained by analyzing capsid images acquired by cryo-EM of complete virions in an earlier study [16]. Therefore, more studies are required to firmly establish whether the capsid has a defined (pseudo-) symmetry or not.

Among the various morphology of PFV viruses, one large population (Class C in Fig 2) representing 57% of the spherical particles has no sign of regular internal ordering and it is also the most variable class in terms of dimensions. The smallest members of this class cannot accommodate the 30 nm capsid of the mature particles and may constitute Env-only virus-like particles consistent with the role of Env in budding [23].

### *In cellulo* replication of PFV

We used high pressure freezing and cryo substitution to analyze thin sections of PFV infected cells by ET. We observed viruses in various states and cellular localization. The landmark for each virus is the capsid, which appears as a dense round material in the cell. Capsids were present with or without viral membrane as reported before [15,21]. In addition, we found viruses budding from the plasma membrane, which have an intermediate shell of density between the capsid and the viral membrane. The same topology is found in released particles by cryo-ET and cryo-EM confirming that these particles are the mature form of PFV and that maturation occurs in the cell before virus egress.

We also observed capsids accumulating in intracellular membrane tubes. Although PFV capsid assembly was reported to take place at the pericentriolar region [15], we provide evidence that capsids accumulate in a membrane compartment whose origin is currently unknown. We suggest that they are immature unprocessed capsids, because of their slightly larger radius compared to the capsid at the plasma membrane (S1 Fig). Notably the intermediate shell of density present in mature capsids [16] is absent and a closer interaction of the N-terminal domain of Gag with the core capsid could explain the larger capsid radius. Because they lack glycoprotein they are most likely not related to budding into intracellular vacuoles as observed before [21]. However, the nature of these potentially capsid-like structures has to be confirmed by further experiments as it cannot be ruled out that they are only indirectly related or completely unrelated to PFV. For instance, virus-related small vesicles have been recently reported during Tick-Borne Encephalitis Virus (TEBV) infection [39]. In contrast, these TEBV related vesicles were variable in size and not arranged regularly as in the case of PFV infection.

### PFV glycoprotein structure

The glycoprotein plays a central role in entry and virus budding [1]. Cryo-ET combined with sub-tomogram averaging is a well tailored method to study glycoprotein *in situ* (embedded in the viral membrane). Several such structures have been determined to medium resolution for different enveloped viruses (up to 20Å for HIV, SIV Env [40]). Here we used a different strategy and acquired 2D images on a direct electron detector by cryo-EM. We took advantage of the high density of glycoprotein at the surface of PFV as well as its symmetrical organization [14] and advanced automatic particle picking to reconstruct *in situ* PFV glycoprotein to ~9 Å resolution. We propose that PFV Env is a class I glycoprotein due to the two predicted coiled coil regions in the gp48^TM^ fusion protein subunit and consistent with this prediction, we identify three rods of density centrally located in the structure which we interpret as a coiled coil region, a hall mark of class I fusion glycoproteins [41–48]. Furthermore, the maps show clear density for three TMHs derived from gp48^TM^ and three from gp18^LP^. The central gp48^TM^ TMHs are in close proximity to interact with each other and can be therefore considered to exert the role of a trimerization domain. Because of the importance of the transmembrane region for membrane fusion [49], it will be interesting to study PFV Env gp48^TM^ induced fusion due to the presence of the gp18^LP^ transmembrane region. Current models of membrane fusion suggest that the trimeric TM region has to come apart to rearrange the fusion protein to catalyze membrane fusion [50,51]. The extra gp18^LP^ TMHs may prevent the dissociation of the gp48^TM^ TMHs and the TM “complex” may therefore move as a block during fusion. In line with the hypothesis that gp18^LP^ affects fusion, mutations within its cytoplasmic region strongly enhance the fusogenic activity of such mutant glycoproteins [9].

An unusual feature of PFV Env is the physical interaction of glycoproteins with each other on the surface of the viral membrane to form intertwined hexagonal networks [14]. The interaction might be mediated by the extracellular part of gp18^LP^ and/or parts of gp48^TM^, which could act as a spacer between glycoproteins. While this topology is not systematic, it seems to be the preferred one. Clustering of the glycoproteins on the surface has been reported for a number of other viruses having class I spike-like glycoproteins such as ASLV Env [52], Influenza hemagglutinin [53], and class III HSV gB [54]. In all these examples, the glycoproteins rather form patches or clusters arranged without symmetry or regular pattern. It has been suggested that the concentration of glycoprotein on the virus surface may confer an advantage for the virus to more efficiently recognize and attach to cellular receptors. Bunyaviruses, having class II glycoproteins [55], are known to have local or global symmetrical arrangement of glycoprotein on the viral membrane [56–58]: trimeric, tetrameric and icosahedral organization have been reported but none of these resembles PFV's. Clusters of Env glycoproteins have been shown to be the preferred site of host cell interaction and thus entry for HIV-1 [59] and it is therefore likely that PFV Env arrangement in hexameric clusters confers also an advantage for host cell entry. The cellular receptor(s) essential for PFV Env-mediated membrane fusion is/are currently unknown but must be ubiquitously expressed molecule(s) due to the large range of permissive cells. Therefore the hexagonal arrangement of Env could enhance low affinity receptor interactions by increased avidity.

In summary our structural analysis of isolated PFV and of PFV infected cells provides new medium resolution insights into the structural proteins Gag and Env, two important players in the PFV life cycle and will thus help to further develop PFV as a gene therapy transfer vector.

## Material and Methods

### Recombinant plasmid DNAs

For production of replication-deficient, wild type PFV vector supernatant a 4 component PFV vector system, consisting of the expression-optimized packaging constructs pcoPG4 (PFV Gag), pcoPE (PFV Env), pcoPP (Pol), and the enhanced green fluorescent protein (eGFP)-expressing PFV transfer vector puc2MD9 was used, which has been described previously [8,60,61]. Mutant PFV vector particles deficient in nucleic acid incorporation (iNAB) were generated using the PFV Gag packaging construct pcoPG4 GR R/A whereas particles deficient in PFV Env fusion (iFuse) were generated using the PFV Env packaging construct pcoPE32 instead of the respective wild type packaging constructs [12,62]. The CMV-driven proviral expression vector pczHSRV2 (wt), described previously [63], was used for production of replication-competent PFV supernatants.

### Transfection and virus production

The human kidney cell line 293T [64] was cultivated in Dulbecco’s modified Eagle’s medium (DMEM) supplemented with 10% heat-inactivated fetal calf serum and antibiotics. Cell culture supernatants containing recombinant viral particles were generated by transfection of 293T cells with the corresponding plasmids using polyethyleneimine (PEI) as described previously [7,8]. For subsequent Western blot analysis the supernatant generated by transient transfection was harvested, passed through a 0.45-μm filter and centrifuged at 4°C and 25,000 rpm for 3 h in a SW32Ti rotor (Beckman) through a 20% sucrose cushion. The particulate material was resuspended in phosphate-buffered saline (PBS). For cryo-EM analysis, viral particles were produced in serum-free medium and a further concentration step using Amicon Ultra 0.5 ml 100K concentrators was included following the first concentration by ultracentrifugation through 20% sucrose similar as described recently [20].

### Western blot analysis

Cells from a single transfected 100-mm cell culture dish were lysed in detergent-containing buffer and the lysates were subsequently centrifuged through a QIAshredder column (QIAGEN). Protein samples from cellular lysates or purified particulate material were separated by SDS-PAGE on a 10% polyacrylamide gel and analyzed by immunoblotting as described previously [9]. Hybridoma supernatants specific for PFV Env LP (Env LP, clone P3B8-B7), PFV Env SU (Env SU, clone P3E10) or PFV Gag (Gag, clone SGG-1) were employed [13,15,61]. After incubation with a horseradish peroxidase (HRP)-conjugated secondary antibody, the blots were developed with Immobilon Western HRP substrate. The chemiluminescence signal was digitally recorded using a LAS-3000 (Fujifilm) imager and quantified using ImageGauge (Fujifilm).

### Cryo-electron microscopy and tomography data collection

Wt PFV, iNAB and iFuse mutants were all prepared following the same procedure except that wt PFV particles were first inactivated for at least 1 h in 4% paraformaldehyde before being processed. 4 μl of sample containing 10 nm gold beads was applied to 2:1 Quantifoil holey carbon grid (Quantifoil Micro Tools GmbH, Germany) and the grid was plunge frozen in liquid ethane with a Vitrobot Mark II (FEI, the Netherlands). For cryo-ET, the frozen grid was transferred to a FEI F20 FEG cryo electron microscope. Tilt series were recorded at 200kV with FEI tomography at a nominal magnification of 29,000 on a 4 k by 4 k Eagle CCD camera from -60 to +60° in 2° steps for a total dose of ~60 e^-^/Å^2^. The defocus was set between -2 and -8 μm. Tilt series were aligned using gold particles as fiducials, binned two times (final sampling of 7.6 Å/pixel) and tomograms were calculated with IMOD [65]. For visualization, the tomograms were denoised by anisotropic diffusion in IMOD.

For cryo-EM, samples were observed with a FEI Polara at 300 kV. Images were recorded on a K2 summit direct detector (Gatan Inc., USA) in super resolution counting mode. For the iNAB sample, movies were recorded at a nominal magnification of 15,500 (1.32 Å/pixel at the camera level) for a total exposure of 4s and 100 ms per frame resulting in 40 frames movies with a total dose of ~20 e^-^/Å^2^. For iFuse, the magnification was 20,000 (0.97 Å/pixel at the camera level), the total exposure was 6s with frames of 0.2 ms resulting in 30 frames movies with a total dose of ~25 e^-^/Å^2^. 230 and 297 movies were manually recorded with Digital Micrograph (Gatan Inc., USA) for iNAB and iFuse PFV respectively.

### Sub tomogram averaging of the glycoprotein

Data interpretation and processing were done with IMOD and PEET [66,67]. The protocol described below was applied for subtomogram averaging of glycoproteins of wt PFV, iNAB and iFuse mutants. The center and radius (defined as the distance from the center to the outer margin of the viral membrane) of each virus was defined in 3dmod. Initial centers for the glycoprotein sub volumes were defined on each virus surface on a regular grid (defined by the spacing between the centers of the neighbor subvolumes and the distance between the subvolume and virus centers) with seedSpikes in PEET. Two of the three Euler angles were estimated based on the spike orientation on the virus surface with spikeInit in PEET. Sub tomogram averaging was done in PEET. The initial reference was an average of all the sub volumes. In the first iterations, no symmetry was enforced but a cylindrical mask was applied to eliminate contributions from the neighbor spikes. The resulting asymmetric reconstruction clearly shows 3-fold symmetry (which was also seen in raw tomograms). C3 was then enforced in the subsequent steps. At that stage, the subtomogram positions were checked manually in 3dmod and sub volumes that converged at positions where there was no glycoprotein or at the same position as other subvolumes were discarded. A final 3D reconstruction of 40^3^ voxels was calculated with this new set of “clean” subvolumes using a soft edged mask around the reference. To obtain reconstruction of the hexagonal network, few more iterations were done without any masking and by increasing the reconstructed volume to 70^3^ voxels. The final 3D models include 2000, 3000 and 3000 subvolumes for wt, iNAB and iFuse PFV respectively. The final resolutions calculated by Fourier Shell Correlation between two half reconstructions were 3.2, 2.9 and 2.8 nm at FSC = 0.5 for respectively wt, iNAB and iFuse PFV.

### Glycoprotein 3D reconstructions from 2D images acquired by cryo-EM

Images were first motion corrected with unblur [68]. From the 3D reconstruction of a glycoprotein hexagonal network obtained by subtomogram averaging (see above), we extracted one hexagon of six trimers and centered it on its 6-fold axis. The resulting reconstruction was used as a template for automatic particle picking using the Fast Projection Matching (FPM) method developed in the lab [69]. To speed up calculation, the raw micrographs were binned by a factor of 4 (5.28 Å/pixel) and 6 (5.82 Å/pixel) for iNAB and iFuse samples respectively. Masks were designed manually around each viral particle to further restrict the area where the automatic picking of glycoproteins was performed. For this initial automatic picking by template matching, the resolution was limited to 3 nm. For each micrograph, only particles having cross correlation with the reference higher that the mean correlation of all the particles were kept. The output coordinate files from FPM were converted to boxer [70] one (.box) and imported in RELION [71] where all subsequent steps were done with two-times binned images (final sampling of 2.64 and 1.94 Å/pixel for iNAB and iFuse respectively). CTF estimation, particle extraction (in boxes of 160 and 218 pixels) for iNAB and iFuse respectively) and preprocessing were done in RELION. The data sets were first cleaned by 2D classification and only classes showing clear glycoprotein network were kept. A first 3D autorefine was then done using the model obtained by subtomogram averaging as an initial reference (low pass filtered to 60 Å) and imposing C6 symmetry. The obtained 3D model was used as input for a 3D classification with 5 classes and C6 symmetry. The particles belonging to the classes showing the best resolved features were used to do another 3D autorefine. The resulting 3D reconstruction showed much improved features than the subtomogram 3D model. Therefore, we did another round of automatic picking with FPM using the latter model calculated with RELION as a reference and extending the resolution limit from 3 to 2 nm. The new coordinate files were imported again in RELION and the same procedure described above was repeated (2D classification, 3Dautorefine, 3D classification and 3D autorefine) leading to new improved maps. We noticed that masking away the viral membrane by keeping only the densities corresponding to the extracelllular domains of the glycoprotein improved the alignment and quality of the 3D reconstruction. Following the last 3D autorefine, we therefore decided to do a focused 3D classification within a mask containing only the extracellular domains of the spikes and their TM regions (3 classes total without alignment). This leads to one class having much sharper details. We used the particles from this class to do a last 3D autorefine. The final reconstructions of hexagonal assemblies include 5541 out of 29659 and 5543 out of 25680 particles for respectively iNAB and iFuse and have resolutions of 11.7 and 9.8 Å at FSC = 0.143.

### Three fold symmetrization of the trimer

Each hexagonal assembly consists of six identical trimers and each one of them has an additional 3-fold symmetry that was not yet applied. The center of one trimer was defined in 3dmod and volumes of 100^3^, 136^3^ voxels for iNAB and iFuse dataset respectively were extracted from the two half unfiltered maps calculated by RELION in the last 3Dautorefine run. The two volumes were then aligned such as their 3fold axis lies parallel to the Z axis with e2align3D.py from EMAN2 [69]. The two aligned volumes were then 3-fold symmetrized before being post processed in RELION with a mask to remove the contribution from neighbor spikes. This leads to an improvement of the density maps to 9.1 and 8.8 Å for iNAB and iFuse dataset respectively at FSC = 0.143.

### 2D Classification of the capsid alone

Intact capsids from the iFuse mutant were picked manually from the same cryo-EM micrographs (binned 6 times to 5.82 Å/pixel) used for the glycoprotein reconstruction with boxer [70]. Particles were extracted in boxes of 130 pixels with RELION and 2D classification in RELION was performed which resulted in the isolation of a subset of 481 intact and regular particles. These particles were assembled in a stack, low pass filtered to 30 Å and further 2D classified in 5 classes by Multi variate Statistical Analysis in IMAGIC [72].

### Density maps interpretation

The 3D reconstructions of single trimer were sharpened with Bfactors of -780 Å^2^and -730 Å^2^ for iNAB and iFuse dataset respectively in RELION. Density maps visualization, segmentation and figure preparation were done with CHIMERA [73]. PFV Env amino acid sequence was submitted to the GeneSilico Metaserver (https://genesilico.pl/meta2 [74]) to predict the number and position of transmembrane helices. No structural homologues was found by the metaserver. Coiled coil prediction in PFV gp48 was done either with Coils [26] or Multicoils [25].

### Cellular electron microscopy on PFV infected cells

Human fibrosarcoma cells HT1080 were cultivated in Dulbecco’s modified Eagle’s medium (DMEM) supplemented with 10% heat-inactivated fetal calf serum and antibiotics. After 48 to 72h, cells were infected at a MOI of 10 and harvested 24 to 48 hours post infection. The cells were fixed and the virus inactivated with 4% paraformaldehyde. The cells were gently detached, pelleted at 500 g and most of the supernatant removed to obtain a thick cell slurry. The cell suspension was loaded in 100 μm deep gold carrier and cryo immobilized by high pressure freezing with a HPM100 (Leica). The carriers were then transferred in an AFS2 device (Leica) maintained at -120°C. The temperature was slowly raised to -90°C before starting the cryo-substitution protocol (adapted for Hawes et al. [75]). The freeze substitution cocktail (0.5% UrAc, 5% EtOH, 5% H20 in acetone) was added for 4h at -90°C. The temperature was then raised at 20°C/h to -50°C. Samples were washed with Ethanol (1h) and then infiltrated with increasing concentrations (33, 50, 66%; 1 h each) of Lowicryl (HM20) in ethanol followed by three incubation in 100% HM20 (2x1h + 18h). UV polymerization was initiated for 48h at -50°C, the temperature was then raised to 18°C (10°C/hour) and polymerization continued for 40 h. Sections (100 to 300nm thick) were obtained with a UC7 (Leica) ultramicrotome and post-stained with 5% UrAc and 1% Lead Citrate. For electron tomography, 10 nm gold beads were added to both sides of the sections. The grids were loaded in a F20 (FEI, the Netherlands) FEG electron microscope. Tilt series were recorded at 200kV with FEI tomography at a nominal magnification of 29,000 on a 4 k by 4 k Eagle CCD camera from -60 to 60° in 3° steps. The defocus was set around -1μm. Tilt series were aligned using gold particles as fiducials, binned two times (final sampling of 7.6 Å/pixel) and tomograms were calculated with IMOD [65]. Segmentation was done in 3dmod as well.

## Supporting Information

S1 Fig Electron microscopy of PFV infected cells. A- Low magnification view of a HT1080 cell infected with PFV prepared by high pressure freezing and cryo substitution. The rectangular box delimits a typical membranous region where capsid-like objects contained in membrane delimited tubes are found. B- 0.8 nm thick section through a tomogram of a region similar to the one shown in A. C- Segmentation of the tomogram in B. Capsids are in cyan and the membranes are represented by transparent green cylinders. D- Radial density profile of capsids in tube (green line) and at the plasma membrane (red line) calculated from averages of n subvolumes (n = 90 and 30 for the capsids in tube and at the plasma membrane respectively).
(TIF)

S2 Fig Production of the various samples used in the study. A- Schematic of PFV Env and Gag domains. The numbers indicate amino acids, the arrows point at cleavage sites. Light grey-shaded regions in PFV Env and Gag represent the Trans Membrane Helices (TMHs) and the Glycine Arginine rich regions (GRI to III) respectively. The dark grey-shaded region in PFV Gag represents p3^Gag^. B- Western blot analysis of wt PFV and of the mutants iNAB and iFuse. Left panel, detection of viral proteins in cell lysates and right panel, detection of viral proteins in purified virions. The identity of the viral proteins is indicated on the right.
(TIF)

S3 Fig Cryo-electron tomography of PFV. A- wt PFV virus dimensions. Histogram of spherical virion's radius colored according to virus morphology (class A: blue, class B: green and class C: red). B- Slice through a tomogram of PFV iFuse mutant showing similar morphology to wt viruses. C- FSC curves for the 3D reconstructions of the various PFV glycoproteins obtained by subtomogram averaging. D, E- 0.8 nm thick tomographic slices perpendicular to the glycoprotein long axis from the wt PFV confirming they are trimeric (the two black arrows point at two such instances).
(TIF)

S4 Fig Side by side comparison of 3D reconstructions obtained for the glycoprotein of the iFuse (left column) and iNAB (right column) mutants by cryo-EM. A & D- Field of view of iFuse and iNAB virus. B & E- Isosurface representation of the sharpened 3D reconstruction obtained for one trimer colored according to local resolution. C & F- Grayscale section through the 3D reconstructions obtained for iFuse and iNAB glycoproteins (side view as in B & E). G- FSC plots for the hexagonal assembly (6-fold symmetry applied) and single spike (additional 3-fold symmetry applied) 3D reconstructions obtained for the iNAB and iFuse mutants. H- FSC plot between the iNAB and iFuse single spike 3D reconstructions illustrating how similar they are. Scale bar in B, C and E, F is 50 Å
(TIF)

S5 Fig Change of appearance of PFV spike 3D reconstruction with filtering to lower resolution. A–C Side views (right column: slab through the center of the structure) of the unsharpened PFV iFuse spike filtered to 9 (A), 15 (B) and 20 Å (C). As the resolution is lowered the overall shape and the large space (indicated with a white star) of the map are unaltered. The large space observed in the map is therefore not likely fully occupied by protein density which would fade into the background at low resolution and get resolved at higher resolution. This artifact has been described by Bartesaghi et al. [44]
(TIF)

S1 Table Summary of data acquisition and analysis for cryo-EM and subtomogram averaging (TIF)

S1 Movie HT1080 cells infected with PFV. The tomogram depicts an area where capsid-like objects are contained within membrane-delimited tubes (relates to S1 Fig).
(AVI)
