# Supplementary figures and images for: Cryo-electron Microscopy Structure of the Native Prototype Foamy Virus Glycoprotein and Virus Architecture
Source: PLoS Pathog. 2016 Jul 11;12(7):e1005721. doi: 10.1371/journal.ppat.1005721 (PMC4939959; doi:10.1371/journal.ppat.1005721)

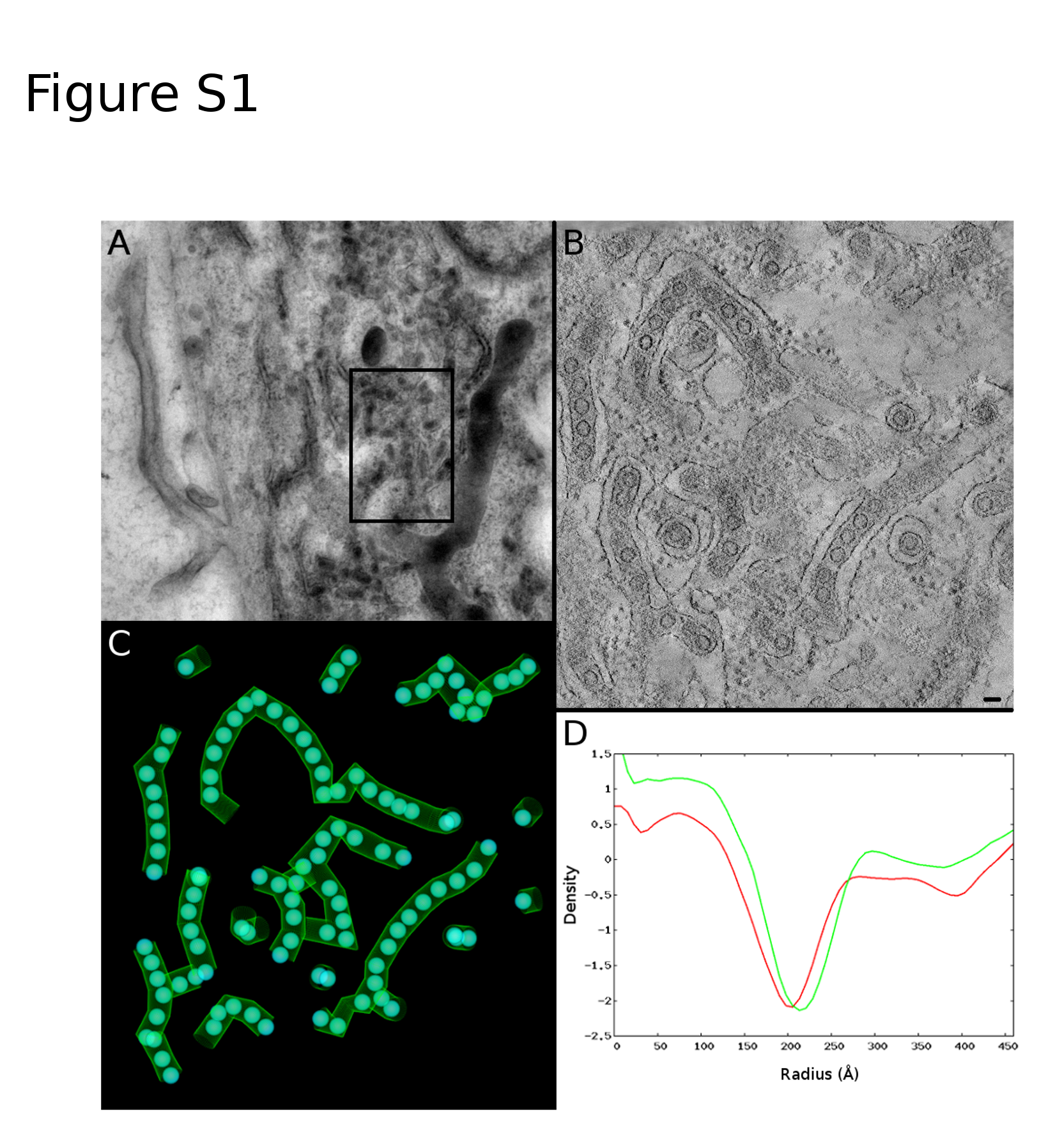

Supplement: S1 Fig — A- Low magnification view of a HT1080 cell infected with PFV prepared by high pressure freezing and cryo substitution. The rectangular box delimits a typical membranous region where capsid-like objects contained in membrane delimited tubes are found. B- 0.8 nm thick section through a tomogram of a region similar to the one shown in A. C- Segmentation of the tomogram in B. Capsids are in cyan and the membranes are represented by transparent green cylinders. D- Radial density profile of capsids in tube (green line) and at the plasma membrane (red line) calculated from averages of n subvolumes (n = 90 and 30 for the capsids in tube and at the plasma membrane respectively). (TIF) [file ppat.1005721.s001.tif]

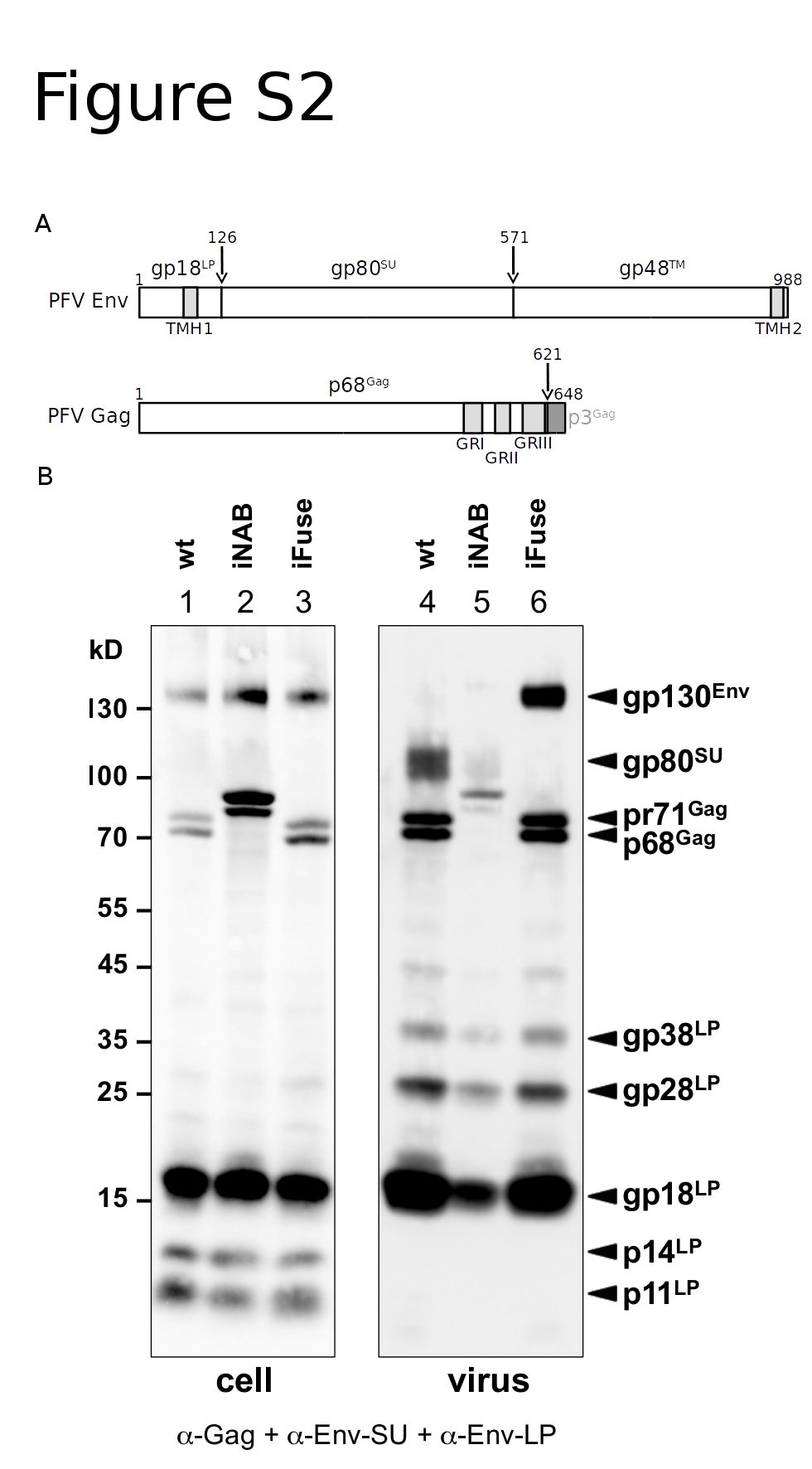

Supplement: S2 Fig — A- Schematic of PFV Env and Gag domains. The numbers indicate amino acids, the arrows point at cleavage sites. Light grey-shaded regions in PFV Env and Gag represent the Trans Membrane Helices (TMHs) and the Glycine Arginine rich regions (GRI to III) respectively. The dark grey-shaded region in PFV Gag represents p3Gag. B- Western blot analysis of wt PFV and of the mutants iNAB and iFuse. Left panel, detection of viral proteins in cell lysates and right panel, detection of viral proteins in purified virions. The identity of the viral proteins is indicated on the right. (TIF) [file ppat.1005721.s002.tif]

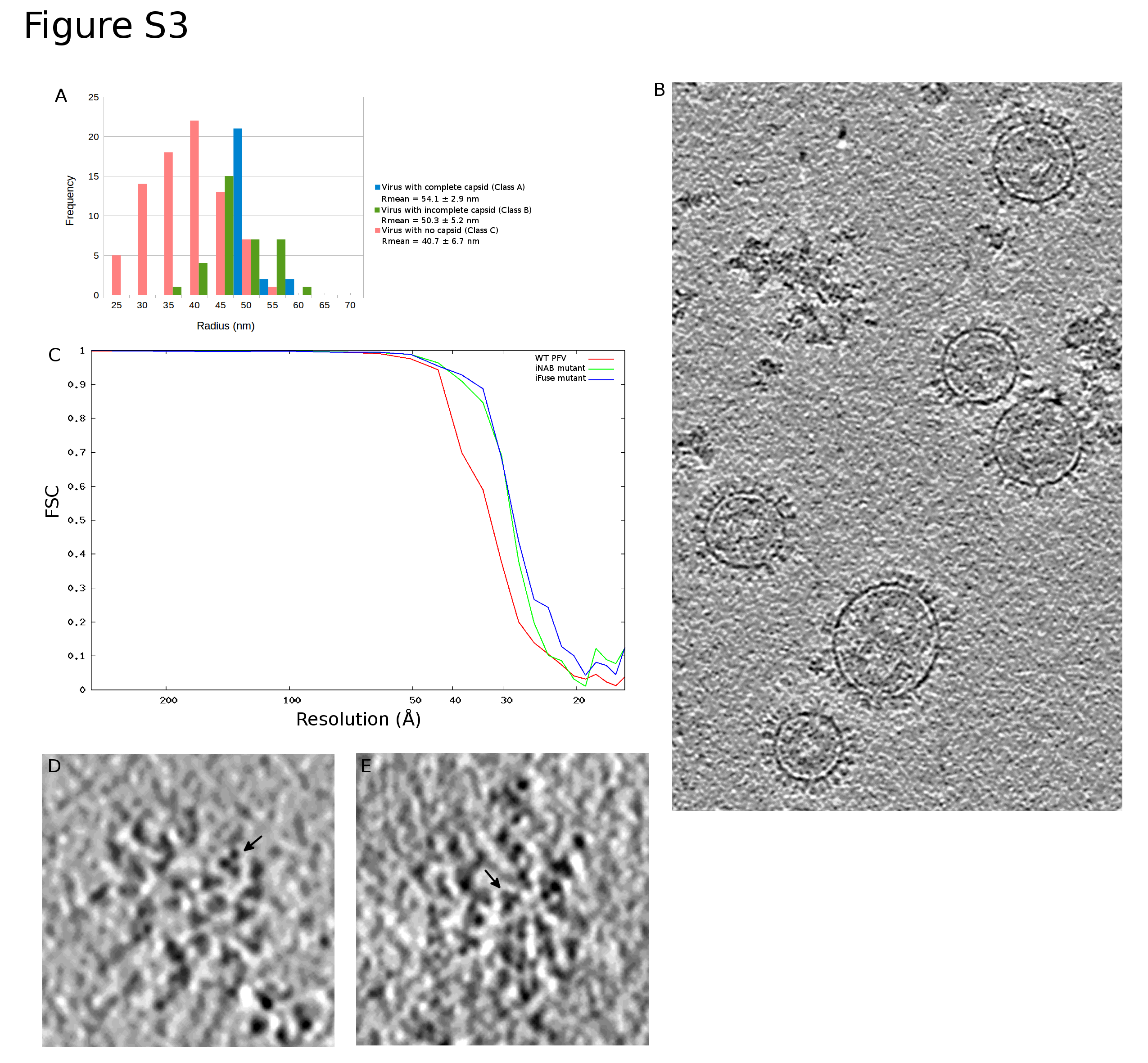

Supplement: S3 Fig — A- wt PFV virus dimensions. Histogram of spherical virion's radius colored according to virus morphology (class A: blue, class B: green and class C: red). B- Slice through a tomogram of PFV iFuse mutant showing similar morphology to wt viruses. C- FSC curves for the 3D reconstructions of the various PFV glycoproteins obtained by subtomogram averaging. D, E- 0.8 nm thick tomographic slices perpendicular to the glycoprotein long axis from the wt PFV confirming they are trimeric (the two black arrows point at two such instances). (TIF) [file ppat.1005721.s003.tif]

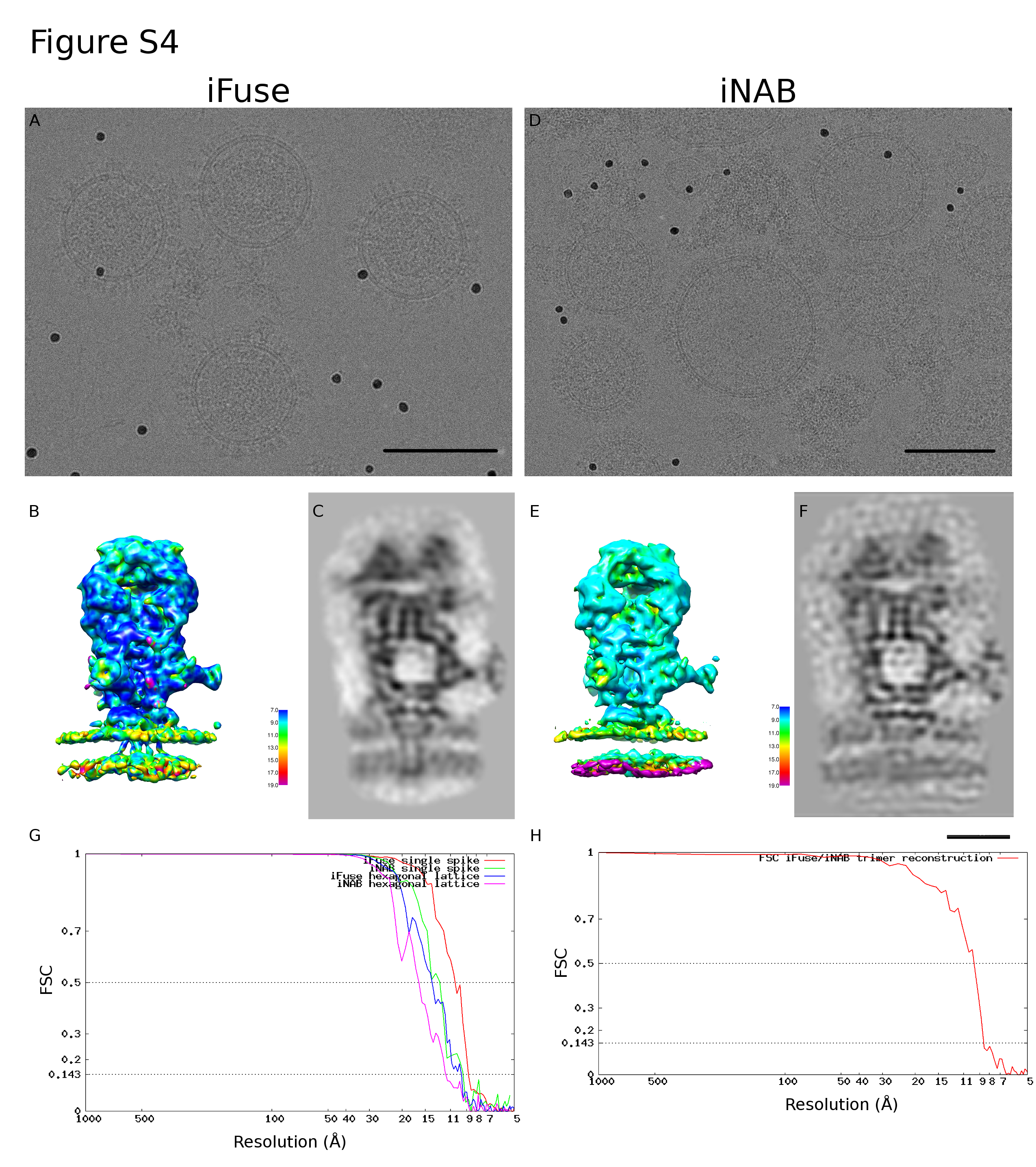

Supplement: S4 Fig — A & D- Field of view of iFuse and iNAB virus. B & E- Isosurface representation of the sharpened 3D reconstruction obtained for one trimer colored according to local resolution. C & F- Grayscale section through the 3D reconstructions obtained for iFuse and iNAB glycoproteins (side view as in B & E). G- FSC plots for the hexagonal assembly (6-fold symmetry applied) and single spike (additional 3-fold symmetry applied) 3D reconstructions obtained for the iNAB and iFuse mutants. H- FSC plot between the iNAB and iFuse single spike 3D reconstructions illustrating how similar they are. Scale bar in B, C and E, F is 50 Å (TIF) [file ppat.1005721.s004.tif]

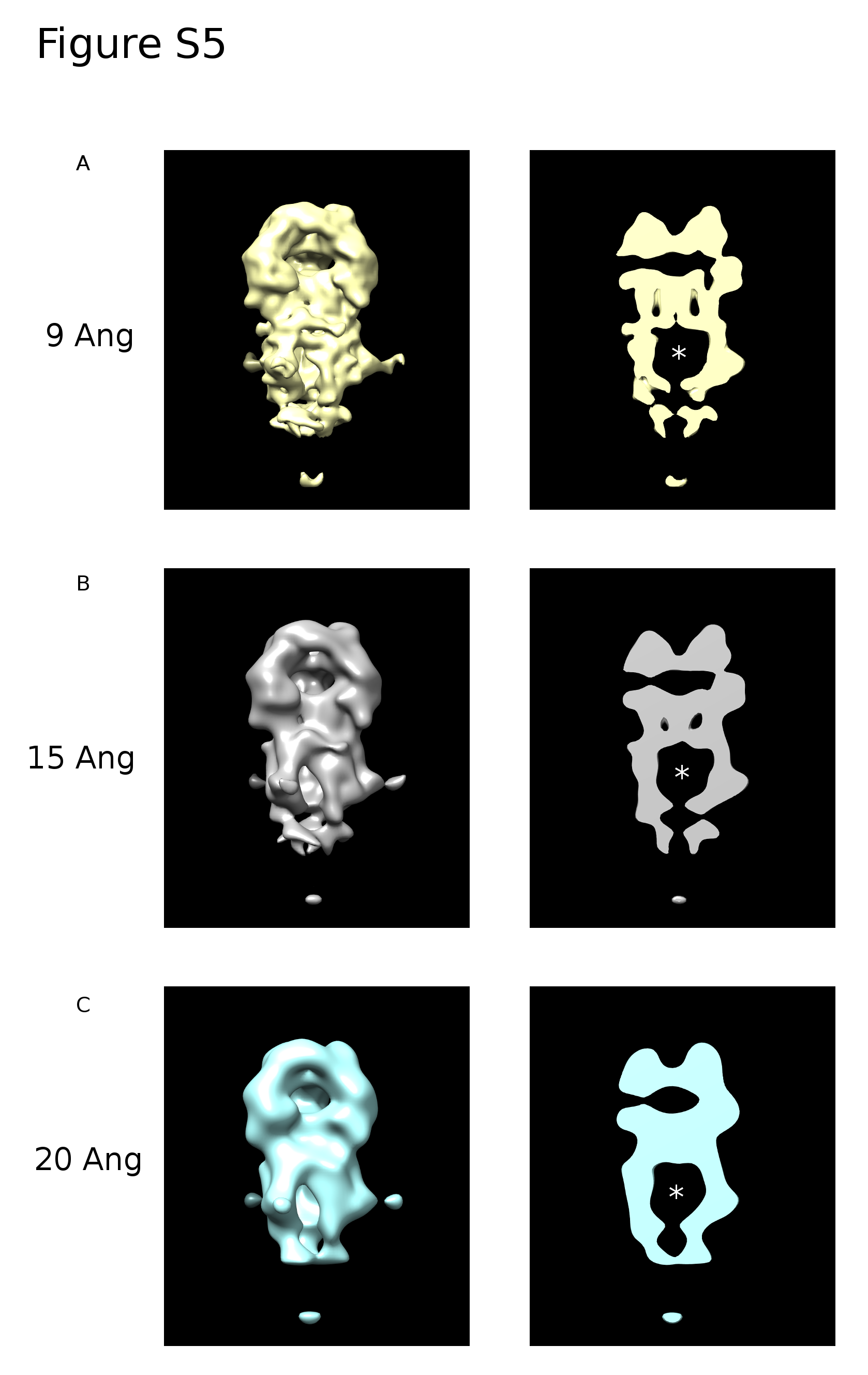

Supplement: S5 Fig — A–C Side views (right column: slab through the center of the structure) of the unsharpened PFV iFuse spike filtered to 9 (A), 15 (B) and 20 Å (C). As the resolution is lowered the overall shape and the large space (indicated with a white star) of the map are unaltered. The large space observed in the map is therefore not likely fully occupied by protein density which would fade into the background at low resolution and get resolved at higher resolution. This artifact has been described by Bartesaghi et al. [44] (TIF) [file ppat.1005721.s005.tif]

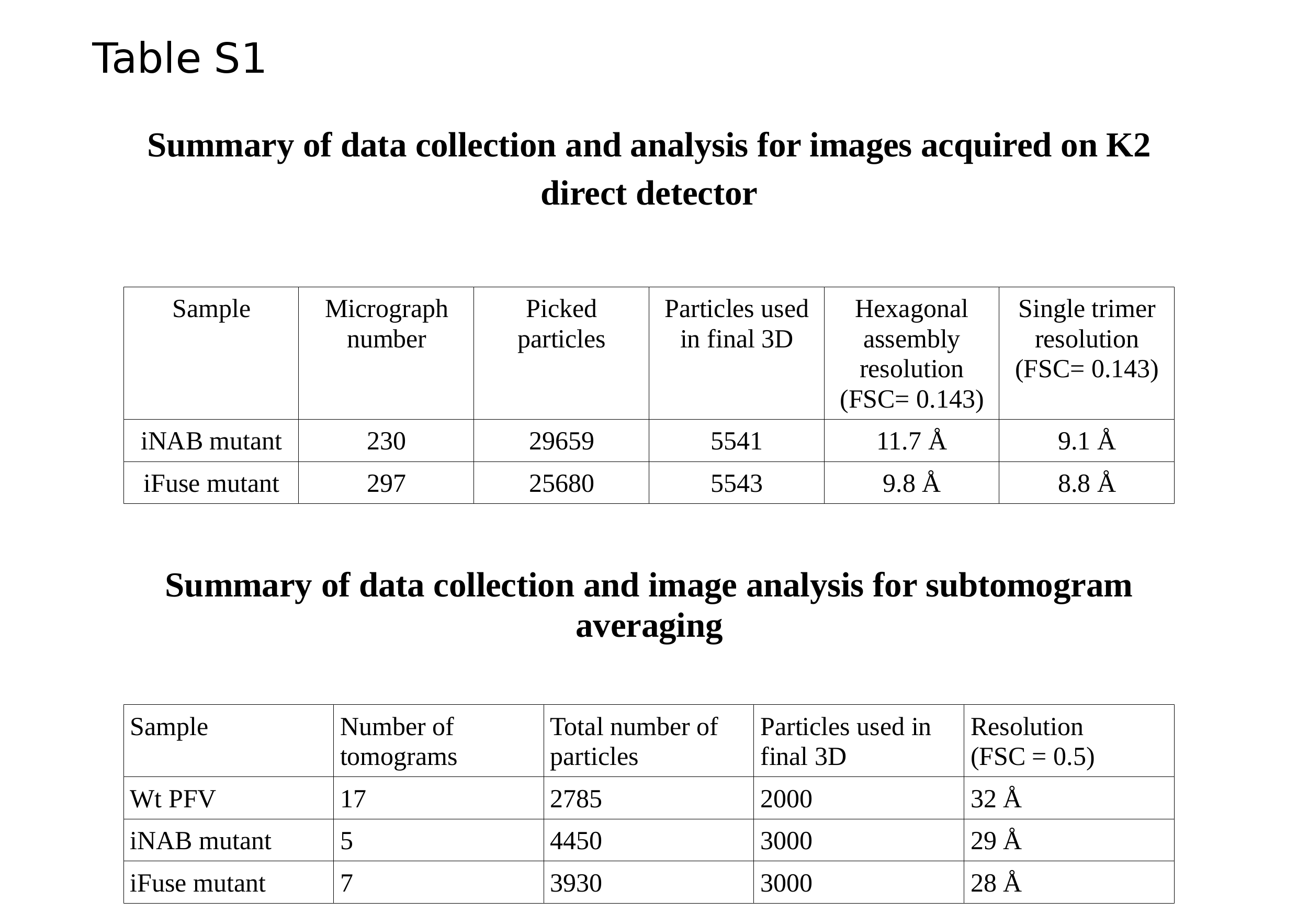

Supplement: S1 Table — (TIF) [file ppat.1005721.s006.tif]
